# Histological changes caused by meclofenamic acid in androgen independent prostate cancer tumors: evaluation in a mouse model

**DOI:** 10.1590/S1677-5538.IBJU.2013.00186

**Published:** 2015

**Authors:** Iván Delgado-Enciso, Alejandro D. Soriano-Hernández, Alejandrina Rodriguez-Hernandez, Héctor R. Galvan-Salazar, Daniel A. Montes-Galindo, Rafael Martinez-Martinez, Laura L. Valdez-Velazquez, Rafael Gonzalez-Alvarez, Francisco Espinoza-Gómez, Oscar A. Newton-Sanchez, Agustín Lara-Esqueda, Jose Guzman-Esquivel

**Affiliations:** 1 School of Medicine, University of Colima, Colima, México; 2 Instituto Estatal de Cancerología, Servicios de Salud del Estado de Colima, Colima, México; 3 Hospital General de Zona Nº1 del IMSS, Colima, México; 4 Chemical Sciences School, University of Colima, Colima, México; 5 Chemical Sciences School, University of Colima, Colima, México

**Keywords:** Prostatic Neoplasms, Meclofenamic Acid, Therapeutics, Anti-Inflammatory Agents

## Abstract

Meclofenamic acid is a nonsteroidal anti-inflammatory drug that has shown therapeutic potential for different types of cancers, including androgen-independent prostate neoplasms. The antitumor effect of diverse nonsteroidal anti-inflammatory drugs has been shown to be accompanied by histological and molecular changes that are responsible for this beneficial effect. The objective of the present work was to analyze the histological changes caused by meclofenamic acid in androgen-independent prostate cancer. Tumors were created in a nude mouse model using PC3 cancerous human cells. Meclofenamic acid (10 mg/kg/day; experimental group, n=5) or saline solution (control group, n=5) was administered intraperitoneally for twenty days. Histological analysis was then carried out on the tumors, describing changes in the cellular architecture, fibrosis, and quantification of cellular proliferation and tumor vasculature. Meclofenamic acid causes histological changes that indicate less tumor aggression (less hypercellularity, fewer atypical mitoses, and fewer nuclear polymorphisms), an increase in fibrosis, and reduced cellular proliferation and tumor vascularity. Further studies are needed to evaluate the molecular changes that cause the beneficial and therapeutic effects of meclofenamic acid in androgen-independent prostate cancer.

## INTRODUCTION

Prostate cancer (PCa) is a worldwide public health problem and is the first cause of death by cancer in men over fifty years of age (1). The growth of this neoplasia is generally dependent on androgen stimulation, although at a given moment it can proliferate in a hormone-independent manner. Androgen-independent prostate cancers are the most aggressive and difficult tumors to control, causing the majority of deaths by this neoplasia (2–5). For these reasons new treatments that can help PCa patients, especially those with androgen-independent tumor, are necessary.

Recent data show inflammation to be a critical component in the origin, proliferation, and dissemination of different cancers, including PCa (6, 7), and thus the effect of anti-inflammatory drugs, particularly nonsteroidal anti-inflammatory drugs (NSAIDs), has been studied with great interest. Fenamates are a group of NSAIDs that stand out for their strong anti-inflammatory properties upon COX enzyme inhibition. At the same time they are very effective inhibitors of aldo-keto reductases (AKR), especially the AKR1C subfamily members. The inhibition processes of both COX and AKR1C are involved in the NSAID antitumor effect, and thus fenamates show great potential in the treatment of cancer (8, 9).

Of the fenamates studied in PCa, meclofenamic acid is the one with the greatest therapeutic effect (10). It has shown a high degree of cytotoxicity for both androgen-dependent and androgen-independent PCa. In addition, it has been confirmed in a nude-mouse model of human androgen independent prostate cancer that meclofenamic acid at non-toxic doses (10 mg/kg/day/25days) significantly reduces tumor growth, prolongs survival, and is even capable of generating total tumor regression in up to 25% of mice treated (10).

Therefore, it is of interest to study the mechanisms by which meclofenamic acid produces therapeutic effects in PCa. The objective of the present work was to analyze the histological changes caused by meclofenamic acid on PCa tumors (nude mouse model) as a possible first step towards understanding the drug's antineoplastic effects.

## MATERIALS AND METHODS

Prostate cancer cell line PC3 was employed in this study. PC3 does not respond to androgens, glucocorticoids, epidermal growth factor (EGF), or fibroblastic growth factor (FGF) (11). Cell lines were maintained in DMEM medium (Sigma, St. Louis, MO, USA) and supplemented at 10% (v/v) with fetal bovine serum (FBS) (GIBCO). They were incubated at 37°C, 5% CO_2_, and 97% relative humidity. Drug was obtained from SIGMA-ALDRICH (Belgium) with 98% purity. Meclofenamic acid was dissolved in alcohol at 70% to generate a 440mM stock.

### 

#### Nude-Mouse model of human prostate cancer

A xenotransplanted murine model harboring prostate tumors was created by subcutaneous injection of 1X10^6^ prostate PC3 cells at the dorsum. The mouse strain used in these experiments was Foxn1nu (6-to 8-week-old males from Harlan Mexico, Mexico City). Once tumors reached an approximate diameter of 4 mm, mice were divided into two groups. For 20 days, a single application per day of meclofenamic acid at a volume of 100 μL was intraperitoneally administered to one group of mice at doses of 10 mg/kg/day (n=5) and a second group received saline solution (PBS) (n=5). Mice were euthanized one day after the end of treatment (day 21) and tumors were extracted and histologically processed and analyzed. It has previously been reported that treatment with meclofenamic acid for 25 days significantly reduced tumor growth and on occasion produced complete tumor regression (10). The purpose of the present study was not to evaluate treatment effectiveness, and so treatment was given for only 20 days in order to avoid any total tumor regression and consequent tumor histological analysis loss. Animals were handled according to institutional guidelines and to the Mexican Official Norm regulating laboratory animal use (11, 12).

#### Tumor histological analysis

Neutral buffered formalin-fixed tumor tissue was embedded in paraffin. Tissue sections (5 μm) were prepared using a microtome and mounted on slides. Proliferation and angiogenesis markers were evaluated by immunostaining for Ki-67 (clone MIB-1) and CD34 (clone QBEnd 10), respectively, as previously described (13). DAKO (CA, USA) brand antibodies were used.

Proliferation marker was assessed by counting the number of nuclei with positive stain for Ki-67 and total number of cancer cells at x100 magnification in three representative regions of each tumor. Results were expressed as the proportion of cells that stained positive over the total number of cells. In each tumor, microvessel density was assessed by counting the number of microvessels at x400 magnification in three fields with the highest vascularization. Results were expressed as mean number of microvessels per field. For statistical analysis, fifteen tumor data per group were obtained by analyzing three representative regions of each of five tumors per group.

Since it has previously been demonstrated that there can be an increase in fibrosis in prostate tumors after different treatments (14, 15), Masson's trichrome stain was also carried out to detect collagen. A commercial Dakocytomation (Artisan) Kit was used according to the manufacturer's instructions (16). Fibrosis was considered to be 1) focal, when it covered less than 10% of the tumor area and presented as isolated bands, 2) moderate, when it covered 10–75% of the tumor area and presented as localized fibrotic zones, and 3) extensive, if it presented in generalized form covering more than 75% of the tumor area.

### Statistical analysis

After further normal data distribution corroboration (by Kolmogorov-Smirnov test), Student t test was used to compare microvessel density and Ki-67 marker of tumors treated with meclofenamic acid and saline solution. Statistical significance was interpreted at values of P<0.05 using the MedCalc program (version 8.1.0.0 for Windows; Mariakerke, Belgium).

## RESULTS

**Figure 1:**
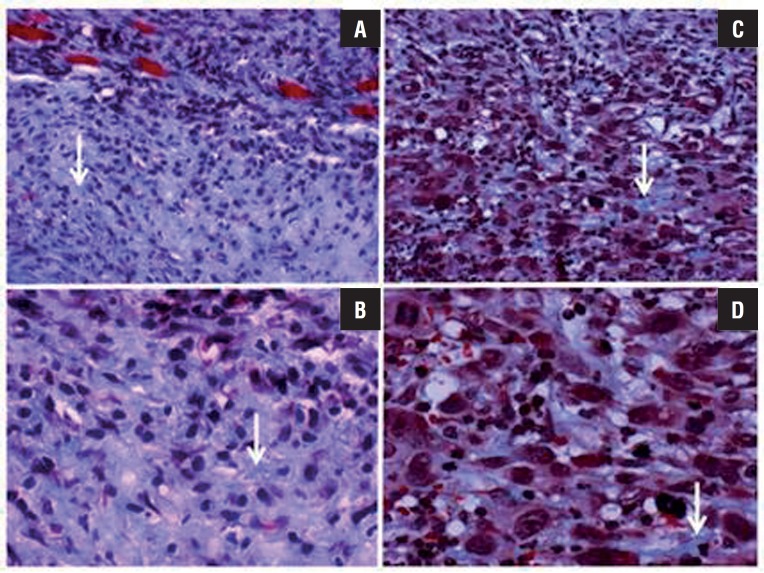
Histological slices representative of PC-3 prostatic tumors stained with Masson´s trichrome. Collagen fibers are blue, as signaled by arrows. Tumors treated with meclofenamic acid (A: X200 and B: X400) present with fibrosis (blue color) clearly covering a large portion of the tumor, whereas tumors treated with PBS (controls) only present with isolated collagen fibers (C: X200 and D: X400). Clear differences in cellularity and nuclear polymorphism are also observed.

At day 21 from beginning of treatment, proliferation and angiogenesis markers (Ki-67 and CD34) and degrees of fibrosis were analyzed in five tumors from the PBS group (control) and from the 10 mg/kg/day meclofenamic acid group. Initially, general histological tumor characteristics were qualitatively analyzed. Tumors treated with PBS had greater hypercellularity, greater nuclear polymorphism, and a greater number of atypical mitoses than the tumors treated with meclofenamic acid (Figure-1). In regard to quantitative analyses supported by immunohistochemical techniques, there was significant reduction in proliferation (Ki-67) and vascularization (CD34) markers (Table-1) in tumors of mice treated with meclofenamic acid.

**Table 1:** Immunohistochemical markers detected in tumors in the nude-mouse model of human prostate cancer.

Marker	Control	Meclofenamate*	P
Ki-67**	22.0±4	13.9±6.6	0.002
CD34***	10.8+2.4	8.5+2.1	0.006

* 10 mg/kg/day dose

** Positive cell percentage (mean±standard deviation)

*** microvessels at x400 magnification per field (mean±standard deviation)

In addition, Masson's trichrome stain showed differences among the groups in regard to degree of fibrosis. Fibrosis was focal in control tumors and was moderate in tumors treated with meclofenamic acid. This result was constant in all five tumors analyzed in each group (Figure-1).

## DISCUSSION

Treatment with meclofenamic acid produces significant histological changes that can be considered beneficial and they are concordant with antitumor effects previously reported for this drug. In an androgen-independent PCa mouse model, Soriano-Hernandez et al. (2011) showed that 25 days of treatment with meclofenamic acid at 10 mg/kg/day significantly reduced tumor growth, prolonged survival and was even able to generate total tumor regression (10). However, the histological mechanisms or changes associated with this beneficial therapeutic effect were not established.

The present study showed that meclofenamic acid caused histological changes denoting less tumor aggression (less hypercellularity, fewer atypical mitoses, and fewer nuclear polymorphisms). It quantitatively confirmed that treatment increased fibrosis, reduced cellular proliferation (Ki-67 reduction) and possibly reduced angiogenesis since it significantly reduced tumor vascularity.

There is evidence in clinical or preclinical trials that NSAIDs can reduce cellular proliferation (determined by means of the Ki-67 marker) in tumors of breast, tongue, cervix, thyroid, and liver (17–21). In different tumor tissues, including PCa, elevated levels of COX-2 have been shown to induce cell proliferation (22, 23), presenting a parallel overexpression of COX-2 and Ki-67 (24, 25). This elevation of COX-2 has also been associated with lower survival rate (24). In addition elevated levels of ARK1C in PCa have been detected and its reduction, through iRNA, causes a decrease in cell proliferation (26, 27). Meclofenamic acid is a potent simultaneous inhibitor of the COX-2 and AKR1C enzymes, which can explain the significant reduction in cellular proliferation in tumors in the mice treated in the present study.

There are different reports regarding the influence of NSAIDs on tumor vasculature. A study in a human breast cancer model showed that COX-2 inhibition by means of celecoxib administration for 7 days significantly reduced microvessel density (28% reduction on average), confirming that angiogenesis had been inhibited (28). Various NSAIDs can inhibit tumor growth by drastically reducing their vascularization (29). This effect is generated when endothelial cell apoptosis is induced (30), or through the reduction of vascular endothelial growth factor (VEGF) levels - one of the principal angiogenesis inducers (31–33). In addition it has been reported that AKR1C3 overexpression promotes angiogenesis and aggressiveness of PCa cells (PC3), suggesting that AKR1C3-inhibition would reduce angiogenesis. This concurs with the significant decrease in vascularity (21% reduction on average) caused by meclofenamic acid (COX-2 and ARK1C inhibitor) in the PCa (PC3 tumors) model in the present study, even though the molecular mechanism responsible for it was not determined.

Also the clear increase in fibrosis in the treated tumors could be a reflection of therapeutic effectiveness. Fibrosis is a parameter that is not often evaluated after PCa treatments. However, it has been observed in tumors in patients with favorable progression after brachytherapy (15) or minimally invasive treatments such as high-intensity focused ultrasound (34). Fibrosis has also been detected in nonmalignant residual prostatic tissue after hormone treatment in humans (35).

In conclusion, meclofenamic acid caused histological changes that indicated decreased tumor aggression, increased fibrosis, and cellular proliferation and vascularity reduction in androgen-independent prostate tumors, lending support to its great therapeutic potential in regard to this neoplasia. Further studies are needed to evaluate the molecular changes that produce meclofenamic acid's histological and therapeutic effects in PCa.
